# Supplementary material for: Study on the effect of digital economy on high-quality economic development in China
Source: PLoS One. 2021 Sep 21;16(9):e0257365. doi: 10.1371/journal.pone.0257365 (PMC8454970; doi:10.1371/journal.pone.0257365)
Supplement: S1 Appendix — (DOCX) [file pone.0257365.s002.docx]

# Appendix I： The process of data standardization and weight determination

This paper evaluates the development level of China's inter-provincial digital economy during the sample period by calculating the digital economy development index of 30 provinces and cities in China (excluding Tibet) (2015-2018). The process of constructing the comprehensive index is as follows.

The first step is the standardization of indicators. As there are differences in the dimensional units of each three-level index, the index should be standardized first. Since the selected indicators are all positive indicators, the reference informatization level index [1] and the network readiness index (NBI), constructed by Harvard University and the World Economic Forum standardize the original data as follows:

$$X_{i}= \frac{V_{i}- V_{min}}{V_{max}- V_{min}}*6+1$$

Among them, $V_{i}$ is the original data of the measure index, $V_{max}$ and $V_{min}$ correspond to the maximum and minimum values of the original data in 30 provinces and cities, respectively. After the data processed by this process, all the index values are between 1 and 7, and the higher the value, the higher the level of the index. However, the current calculation formula is compared in different years, and there is a lack of comparability between different statistical years. Therefore, in order to eliminate the influence of time effect, this paper adopts the method of setting the base year to make the panel data comparable in the base period. This paper sets the measurement index to take 2015 as the base period, and uses the following formula to standardize the data:

$$X_{it}= \frac{V_{it}- V_{min0}}{V_{max0}- V_{min0}}*6+1$$

Where $t$ represents the year of the measure index, $V_{max0}$ and $V_{min0}$ represent the maximum and minimum values of the original data in the base year. After the above preliminary processing, the measure index can be compared between different years, and then the standard normal cumulative probability value of the data is obtained and multiplied by 100, the original data can be transformed into an interval range of 0 and 100. The formula is as follows:

$r_{ijt}=100*\Phi(\frac{x_{ijt}-\bar{x}}{s_{j}}$)

In the formula,$x_{ijt}$ is the data after the initial processing of the third-level index $j$ in the $t$ year of the $i$ province or city, $r_{ijt}$ is the standardized value, $\bar{x}$ is the mean value and $s_{j}$ is the standard deviation of the internal index $j$ in the sample period of 30 provinces and cities, and the function $\Phi(x)$ is the standard normal cumulative probability distribution value of $x$.

The second step is to determine the weight of the index. This paper evaluates the three-dimensional data of time series panel, and the general static comprehensive evaluation method is not applicable. In order to determine the index weight more accurately from an objective point of view, the vertical and horizontal pull-out method is used to determine the weight of each index [2]. This method can not only reflect the change of horizontal dimension in time, but also reflect the difference of each province and city from the vertical dimension, which makes the comprehensive evaluation results of panel data comparable in cross section and time.

For time $t_{k}$ ($k=1,2,\cdot\cdot\cdot,N)$, $N=4$ in this article, and the comprehensive evaluation function is: $y_{i}\left( t_{k} \right)= \sum_{j=1}^{m} w_{j}r_{ij}\left( t_{k} \right)$. Where $m$ is the number of indicators, $w_{j}$is the weight of indicator $j$, which is the core parameter that needs to be determined in the process. The basic principle of the vertical and horizontal unfolding method is to reflect the difference between the evaluated objects on the time series three-dimensional table to the greatest possible extent, which is more in line with the actual situation of the large difference in the level of digital economy development between provinces in China. For $n$ provinces and cities to be evaluated, after standardizing the original data, we can get:

$$\sigma^{2}= \sum_{k=1}^{N} \sum_{i=1}^{n} {[y_{i}\left( t_{k} \right)- \bar{y}]}^{2}= w^{T} \sum_{k=1}^{N} H_{k}w=w^{T}Hw$$

Where $w={(w_{1}, w_{2}, \cdot\cdot\cdot, w_{m})}^{T}$, $H=\sum_{k=1}^{N} H_{k}$ is a symmetric matrix. Then, let $H_{k}=A_{k}^{T} A_{k}$($k=1,2,\cdot\cdot\cdot,N)$, $A_{k}$ be a real symmetric matrix. If $w^{T}w=1$ when $w$ is the (standard) eigenvector corresponding to the maximum eigenvalue $\lambda_{max}(H)$ of matrix $H$, $\sigma^{2}$ takes the maximum value, and the matrix vector corresponding to the maximum eigenvalue $\lambda_{max}\left( H \right)$ is the weight value of index $j$. The weight values of each third-level index are referred to Table 1.

**References**

1. Zhang Bin, Peng Zhidao, Zhao Lei, et al. International comparative research on China’s information development[J]. China CIO News,2017(06):122-125.
2. Guo Yajun. A new dynamic comprehensive evaluation method[J]. Journal of Management Sciences in China,2002(02):49-54.
