# Supplementary material for: Study on the effect of digital economy on high-quality economic development in China
Source: PLoS One. 2021 Sep 21;16(9):e0257365. doi: 10.1371/journal.pone.0257365 (PMC8454970; doi:10.1371/journal.pone.0257365)
Supplement: Attachment — Submitted filename: Review of PLOS one D21 12870.docx [file pone.0257365.s003.docx]

This research examined the dynamic changes of the digital economy in China. The digitalized economy is a new approach, but this research collapses to measure the current variables used in discussing the digital economy. The precise measurements reflect the accurate discussion relating to the literature and theories. For example, the total factor productivity index used as an independent variable reflects the conventional measurement of GDP, which does not describe the rise of the added value of the digital economy. The common problem is that the sharing of the digital economy is not captured yet by the traditional GDP. The authors try to promote a novelty approach in examining the dynamic changes of the digital economy in China, so they should carefully determine the measurement of the variables. In addition, the authors implemented the Solow Model growth as the basis for the development of hypotheses. In my opinion, if the authors appoint the Solow growth model so they should measure the changes in the level of output of TFP in the case of digital economy activities. They implement the C-D function to measure Inter-provincial Total Factor Productivity, and they support the empirical theories and previous results to show the TFP of the digital economy. Does the conventional TFP reflect the shared digital economy of TFP? How do the authors measure? Please support the empirical findings.

The authors also implement Digital Economic Development Index (DEI) as an independent variable, but they do not explain and support previous findings in the literature review. Do the DEI indices reflect the level of capital based on the Solow Model? How to justify in the literature review?

The authors promote the control variables in the model. However, the measurements do not reflect the digitalization of economic activities. For example, the human capital level is measured with the average number of students in colleges and universities per population. How does the previous measurement reflect the employment creation in the digital economy? The comments are similar in implementing general measurements of FIA, IS, OPEN, and RD in contributing to the growth of the digital economy growth.

It is not described what software it was used with. The other is the disturbance caused by the alpha parameter. This is because the alpha parameter is included in the Cobb-Douglas (CD) function in Section 4.1 (I note incidentally that the formula was not numbered, though it should be), and is included in formulas (2) and (5) and later in the article confused as to which they are referring to. It would be advisable to use a different notation. It was not clear to me how the C-D function was applied, how it relates to the model denoted by formula (4). It would be advisable to use the name Cobb-Douglas instead of C-D first and then the abbreviation.

The English writing needs improvement.

The literature review does not support the determination and measurement of the variables comprehensively.

The manuscript should be even more sufficiently supported by evidence or proper references to work done elsewhere. For this purpose authors could downloads the following published article: <https://doi.org/10.3390/su12114674>, https://doi.org/10.1080/13675567.2017.1393506.

The conclusion does not extend the literature review in term of previous debates and theories.

The citation in the text is not harmonized with the numbering style in the reference.
